# Biomechanical analysis of the posterior bony column of the lumbar spine

**DOI:** 10.1186/s13018-017-0631-y

**Published:** 2017-09-15

**Authors:** Jiukun Li, Shuai Huang, Yubo Tang, Xi Wang, Tao Pan

**Affiliations:** 1 Department of Orthopaedic Surgery, The Sixth Affiliated Hospital of Sun Yat-sen University, 26 Yuancun Er Heng Road, Guangzhou, Guangdong 510655 China; 2 Department of Orthopaedic Surgery, The Second Affiliated Hospital of Guangzhou Medical University, Guangzhou, 510260 China; 3 Department of Pharmacy, The First Affiliated Hospital of Sun Yat-sen University, Guangzhou, 510080 China

**Keywords:** Mechanical properties, Compression test, Lamina, Articular process, Spinous process

## Abstract

**Background:**

Each part of the rear bone structure can become an anchor point for an attachment device. The objective of this study was to evaluate the stiffness and strength of different parts of the rear lumbar bone structure by axial compression damage experiments.

**Methods:**

Five adult male lumbar bone structures from L2 to L5 were exposed. The superior and inferior articular processes, upper and lower edges of the lamina, and upper and lower edges of the spinous process were observed and isolated and then divided into six groups (*n* = 10). The specimens were placed between the compaction disc and the load platform in a universal testing machine, which was first preloaded to 5.0 N tension to eliminate water on the surface and then loaded to the specimen curve decline at a constant tension loading rate of 0.01 mm/s, until the specimens had been destroyed.

**Results:**

Significant differences in mechanical properties were found among different parts of the rear lumbar bone structure. Compared with other parts, the lower edge of the lamina has good mechanical properties, which have a high modulus of elasticity; the superior and inferior articular processes have greater ultimate strength, which can withstand greater compressive loads; and the mechanical properties of the spinous process are poor, and it is significantly stiffer and weaker than the lamina and articular processes.

**Conclusion:**

These data can be useful in future spinal biomechanics research leading to better biomechanical compatibility and provide theoretical references for spinal implant materials.

## Background

Transforaminal interbody fusion (TLIF) was developed as an alternative method for mitigating the risks and limitations associated with posterior lumbar interbody fusion (PLIF). TLIF involves attachment of adjacent vertebral bodies with implants to achieve a fusion effect. Pedicle screw fixation and rod attachment are popular, and bilateral pedicle screw–rod attachment has been widely used. Meanwhile, unilateral pedicle screw procedures have been conducted in PLIF and are a safe and cost-effective method to perform the PLIF procedure [1]. Recent reports show that the minimally invasive posterior lumbar interbody fusion (MI-PLIF) technique is a safe procedure with fewer complications and a high fusion rate [2, 3].

Pedicle screw fixation and rod attachment are popular, but their application has been limited by postoperative complications and high costs [4, 5]. Both unilateral and bilateral pedicle screw–rod attachments in one- or two-segment lumbar spinal fusion have comparable complication rates [6, 7]. Furthermore, if the primary operation with pedicle screw fixation and rod attachment fails, a damaged posterior bony structure makes subsequent rebuilding and stabilization of the spine more challenging [6, 7].

On the basis of the three-column spine theory [8], the spine is divided into three columns: the anterior column, column, and posterior column. When the anterior column is in a pathological state as a result of spondylolisthesis and/or degenerative disc disease, the original spine biomechanical properties change significantly, resulting in spinal load redistribution after attachment. In a pathological state or a fixed state, the function of the anterior column completely diminishes. Thus, the posterior column bears all the pressure, indicating that the posterior structure of the lumbar spine plays a non-negligible role in the reconstruction of the lumbar spine for stability, which provides the theoretical foundation for posterior lumbar fusion [9].

With the advances in biomechanics and material science research, new posterior fixation devices are emerging. Each bone structure of the rear lumbar column can become an anchor point for an attachment device. We have also designed a newly developed shape-memory alloy hook in a TLIF, which can achieve immediate stability [10].

Previous biomechanical studies have shown that the stiffness of the vertebral pedicle is the hardest part, but it just conveys directly the stress of the rear structure to the front vertebral body as a pedicle screw channel and then effectively completes the three-column spine attachment, which has become the gold standard posterior lumbar fusion [11]. However, the pedicle screw placement damages the pedicle structure or the attachment fastness decreased because of osteoporosis and the screw can cause a fatigue fracture, loosening, or even detaching. If the patient does not appear to have lumbar spondylolisthesis or instability, performing the three-column fixation is not necessary. The main role of the facet joints of the spine is anti-rotation and anti-shear, although it is not a major structural function to resist spine compression. However, biomechanical studies show that translaminar facet joint screws [12] can also provide good fixed effect through a facet anchor point. The spinous process, the rearmost bone structure, can achieve spinal canal decompression by interspinous separation or attachment of devices to maintain its height. These devices can reduce disc pressure through their elastic means and thus provide flexible mounting pressure to reduce adjacent segment degeneration [13]. However, the strength and stiffness of the spinous process will decrease and result in spinous process fracture and cause fracture attachment failure in patients with osteoporosis. In addition, these devices damage the interspinous ligament between the spinous process. Studies have shown that complete rear spine ligament complex plays an important role in lumbar stability [8]. There are also studies of a design called Ni-Ti “U” shape-memory alloy devices [10], which anchor to the lamina–lamina, lamina–facet, and transverse process–transverse process, avoiding damaging the posterior lumbar bone structure and protecting the intact spinal motion unit, more in line with the biomechanical characteristics of the spine. In addition, the Harrington’s system unit commonly used in scoliosis also anchors to the lamina–facet. These posterior attachment devices can be designed to anchor to the pedicle, articular process, lamina, transverse, or spinous process; however, whether these different bony structures of the anchor point have different mechanical properties, no relevant literature has been reported.

Previous research has focused on biomechanical performance testing and stress–strain analyses of the spine and intervertebral discs; however, mechanical testing for rear lumbar bone structure is rarely reported. The purpose of this study was to evaluate the differences in the stiffness and strength of each rear lumbar bone anchor point by studying axial compression damage. This information can provide the basic mechanics for a clinical spinal attachment device by determining the optimal implant location, and it can probably be used to develop a trauma model using finite element analysis.

## Methods

### Specimen preparations

**Fig. 1:**
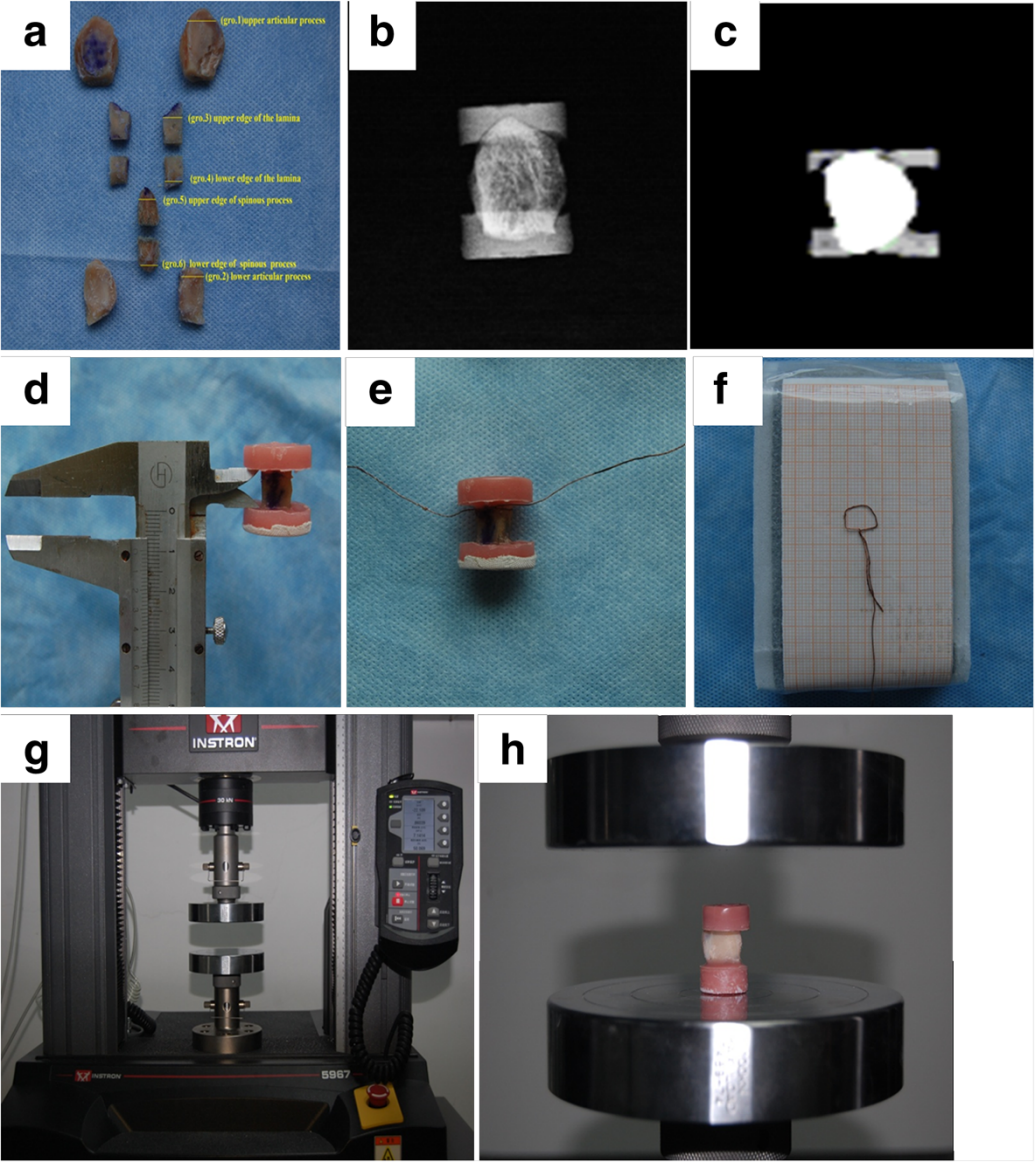
Preparation and measurement of bone specimens. The samples were divided into six groups (**a**). All specimens were tested using imaging studies to rule out pathological abnormalities (**b**, **c**). Measurement of the original length *L*_0_ (**d**). Measurement of force face *S*_0_ (**e**, **f**). Universal testing machine (**g**). The central axis of the specimen coincided with two platen center connections to achieve the shaft load (**h**)

Five adult male lumbar structures were obtained from the Department of Anatomy of Guilin Medical College, and the experiment followed an institutional medical ethics procedure. Individual vertebra, with the paraspinal muscles, interspinous ligament, spinal cord, and other soft tissues removed, were isolated. The L2 to L5 posterior column bone structures were exposed and observed, and the superior and inferior articular processes, upper and lower edges of the lamina, and upper and lower edges of the spinous processes were isolated (Fig. 1a). Using the appropriate anatomical site, these samples were divided into six groups (*n* = 10): the superior articular process (group 1), inferior articular process (group 2), upper edge of the lamina (group 3), lower edge of the lamina (group 4), upper edge of the spinous process (group 5), and lower edge of the spinous process (group 6). Before mechanical testing, all specimens were tested using contact and imaging studies to rule out deformities, degenerative changes, fractures, cancer, osteoporosis, and other pathological abnormalities (Fig. 1b, c). With their upright positions, the cortical axis of all specimens coincided with the central axis of the specimen, and both ends were embedded and fixed with denture powder and denture resin to make compressed samples [14]. The mean length of the exposed bone in the denture powder block was considered as the original length *L*_0_, which was measured thrice using a vernier caliper, with the mean value accuracy of 0.01 mm (Fig. 1d). Due to the irregular geometry of the embedded specimens, these compressed samples could not be processed into standard test pieces. In accordance with engineering mechanics “worst” principle, the smallest cross section of the specimen was surrounded with a small wire (Fig. 1e) and copied to a 1-mm grid paper as the load plane (Fig. 1f) and then measured thrice to derive the mean as the force face *S*_0_. All the specimens were wrapped and sealed with saline-infiltrated gauze and frozen properly at −20 °C for further compression testing.

### Compression testing

**Fig. 2:**
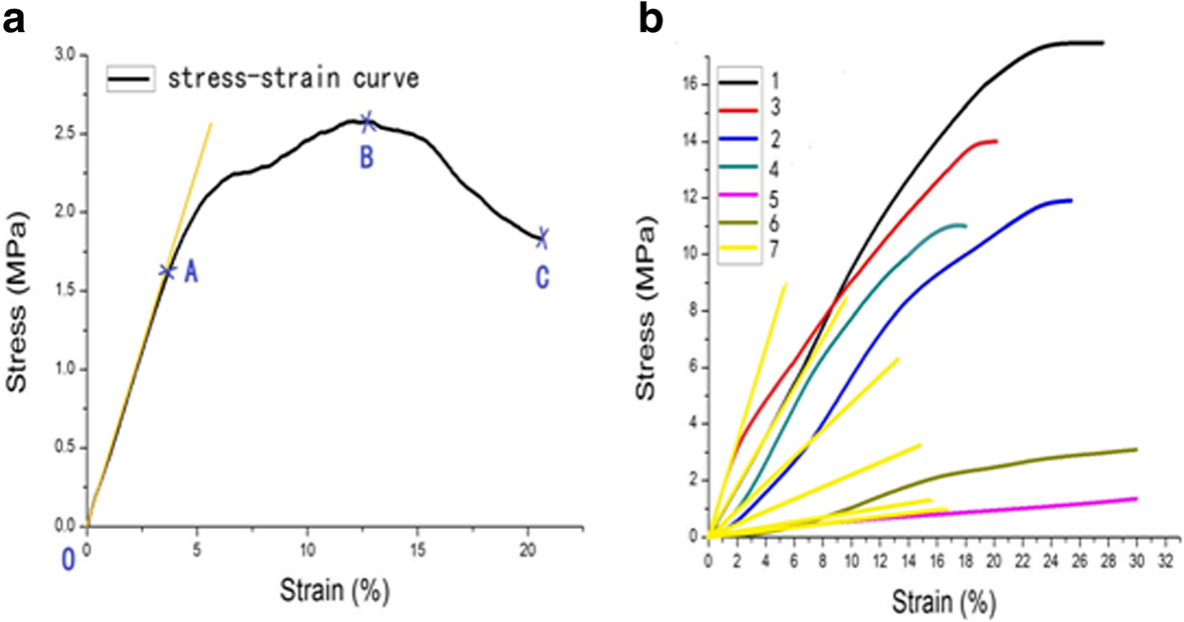
Typical stress–strain curve (**a**). Each group fitting curve (**b**). **a** A typical stress–strain plot is divided into three parts: the elastic, plastic, and breaking phases; the horizontal and vertical axes represent the strain and stress diagrams, respectively. The OA segment is the elastic stage, representing the slope of the curve; the AB segment is the plastic stage, where B is the maximum load corresponding with ultimate strength; and the BC segment is the fracture stage, where C is the point for the specimen to completely fracture. The curve has a good linear relationship (OA), then increased before B and decreased to C. Each group fitting curve is represented in **b**. All curves are stopped at the maximum compressive stress, each set of the curves in the initial stage gradually separated, and no overlap is observed. The slope of the curve in each group set up on the edge of the lamina group, curve peaks corresponding to the maximum pressure on the facet group

Before compression testing, all bone specimens from the −20 °C freezer were naturally thawed at room temperature. Subsequently, the specimens were placed between the compact disc and the load platform (Fig. 1h) in a universal testing machine (Fig. 1g), and the central axis of the specimens was coincided with two platen center connections to achieve a shaft load. All specimens were first preloaded to 5.0 N tension to eliminate water on their surfaces and then loaded to the specimen curve [15] at a constant 0.01 mm/s loading rate [16] until the specimen was destroyed. The loading data were transferred by a mechanical load sensor, and the material testing software program automatically recorded the elastic modulus, stress–strain, and other original data [16]. Typical specimen stress–strain curves were pretreated (Fig. 2a), and the fitting curves of each group are presented in Fig. 2b. Stress was calculated by dividing the applied force for specimens by the measurement of the cross-sectional area. Strain was calculated by dividing the actual deformation of specimens by individual original length. The elastic modulus was determined as the slope of the linear section of stress–strain curve within a strain range of 0.02. The maximum strain, ultimate strength, and maximum load of each specimen were determined when the curve declined, at which the bone specimen began to develop fractures.

### Statistical analysis

Statistical analyses were performed using SPSS 19.0 software. The Kruskal–Wallis test was used between groups; hence, the one-way analysis of variance (ANOVA) was feasibly performed in each group after the normality test with normal distribution was completed. The Levin test showed heterogeneity of variance without assuming homogeneity of the case, and Dunnett-T3 method was used in each group for multiple comparison procedures. *P* < 0.05 was considered as statistically different.

## Results

The descriptive statistics of the mechanical parameters for each group are shown in Table 1. The normal distribution and homogeneity of variance test results are shown in Table 2. Table 3 shows that all groups were analyzed using ANOVA, and a pairwise comparison of each group was made.

**Table 1:** Descriptive statistics of the mechanical parameters of the six groups (*x* ± *s*, *n* = 10)

Group numbers	Elastic modulus (MPa)	Maximum strain (%)	Ultimate strength (MPa)	Maximum load (*N*)
1	62.58 ± 11.07	26.35 ± 3.00	10.99 ± 2.68	1039.14 ± 419.97
2	82.32 ± 16.46	30.82 ± 4.00	15.56 ± 2.76	1170.79 ± 247.59
3	117.20 ± 5.95	22.16 ± 6.09	14.71 ± 3.89	914.48 ± 242.13
4	79.95 ± 13.57	18.39 ± 3.25	11.54 ± 2.62	489.02 ± 77.88
5	47.35 ± 5.80	14.64 ± 2.48	3.47 ± 0.315	196.76 ± 14.27
6	14.86 ± 2.43	14.56 ± 2.23	0.76 ± 0.04	59.52 ± 6.00

*1* superior articular process group, *2* inferior articular process group, *3* upper edge of the lamina group, *4* lower edge of the lamina group, *5* upper edge of the spinous process group, *6* lower edge of the spinous process group

**Table 2:** Results of normal distribution and homogeneity of the variance test of mechanical parameters

Mechanical parameters	Nonparametric	Normality test	ANOVA	Homogeneity test
Elastic modulus (MPa)	*χ* ^2^ = 51.919	*P* = 0.000 < 0.05	*F* = 111.195	*P* = 0.000
Maximum deformation (%)	*χ* ^2^ = 42.519	*P* = 0.000 < 0.05	*F* = 42.528	*P* = 0.000
Ultimate strength (MPa)	*χ* ^2^ = 46.820	*P* = 0.000 < 0.05	*F* = 59.557	*P* = 0.000
Maximum load (N)	*χ* ^2^ = 50.075	*P* = 0.000 < 0.05	*F* = 30.787	*P* = 0.000

The Kruskal–Wallis test prompted significant differences between groups; one-way analysis of variance (ANOVA) was feasibly performed in each group; the normality test conformed to normal distribution (*P* = 0.000); and the Levin test showed heterogeneity of variance (*P* = 0.000)

**Table 3:** Pairwise comparison table of the mechanical parameters of the six groups (Dunnett-T3)

Elastic modulus (MPa)	Ultimate strain (%)	Maximum load (*N*)	Ultimate strength (MPa)
P1–2 > 0.05	P1–2 > 0.05	P1–2 > 0.05	P1–2 < 0.05
P1–3 < 0.05	P1–3 > 0.05	P1–3 > 0.05	P1–3 > 0.05
P1–4 > 0.05	P1–4 < 0.05	P1–4 < 0.05	P1–4 > 0.05
P1–5 < 0.05	P1–5 < 0.05	P1–5 < 0.05	P1–5 < 0.05
P1–6 < 0.05	P1–6 < 0.05	P1–6 < 0.05	P1–6 < 0.05
P2–3 < 0.05	P2–3 < 0.05	P2–3 > 0.05	P2–3 > 0.05
P2–4 > 0.05	P2–4 < 0.05	P2–4 < 0.05	P2–4 < 0.05
P2–5 < 0.05	P2–5 < 0.05	P2–5 < 0.05	P2–5 < 0.05
P2–6 < 0.05	P2–6 < 0.05	P2–6 < 0.05	P2–6 < 0.05
P3–4 < 0.05	P3–4 > 0.05	P3–4 < 0.05	P3–4 > 0.05
P3–5 < 0.05	P3–5 < 0.05	P3–5 < 0.05	P3–5 < 0.05
P3–6 < 0.05	P3–6 < 0.05	P3–6 < 0.05	P3–6 < 0.05
P4–5 < 0.05	P4–5 > 0.05	P4–5 < 0.05	P4–5 < 0.05
P4–6 < 0.05	P4–6 > 0.05	P4–6 < 0.05	P4–6 < 0.05
P5–6 < 0.05	P5–6 > 0.05	P5–6 < 0.05	P5–6 < 0.05

*P* < 0.05 was considered statistically significant

*1* superior articular process group, *2* inferior articular process group, *3* upper edge of the lamina group, *4* lower edge of the lamina group, *5* upper edge of the spinous process group, *6* lower edge of the spinous process group

**Fig. 3:**
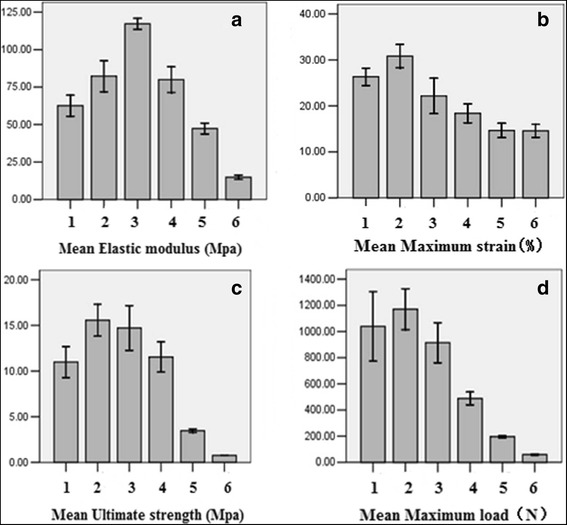
Six groups of histogram specimen mechanical parameters. **a** For each set of elastic modulus, the elastic modulus at maximum is the upper edge of lamina. **b** For each set of elastic strain, ultimate strain at maximum is the inferior articular process. **c** The ultimate strength at maximum is the inferior articular process. **d** The maximum load at maximum is the inferior articular process

Our results demonstrated that, for elastic modulus, the upper edge of lamina had the largest elastic modulus (117.20 ± 5.95 Mpa, Table 1) (Fig. 3a), significantly different among all six groups (*P* < 0.05, Table 3) while there is no significant difference between the inferior articular process and the lower edge of lamina (*P* > 0.05, Table 3). When compared with othe group, the upper and lower edge of the spinous process had smallest elastic modulus vaule, significantly lower than othe groups (*P* < 0.05, Table 3).

The inferior articular process had the ultimate strength maximum value (15.56 ± 2.76 MPa, Table 1) (Fig. 3c), and no significant difference was found in the upper edge of the lamina (*P* > 0.05, Table 3). However, the edge of the lamina ultimate strength (15.56 ± 2.76 MPa, Table 1) was greater than the inferior articular process (10.99 ± 2.68 MPa, Table 1). The inferior articular process had a higher ultimate strength value than the upper edge of the lamina (*P* < 0.05, Table 3). The maximum load of the upper and lower edges of the spinous process was significantly lower than that of each group (*P* < 0.05, Table 3).

For maximum load, no significant difference between the superior and inferior articular processes (*P* > 0.05, Table 3), among which the inferior articular process carried the maximum capacity (1170.79 ± 247.59 N, Table 1) (Fig. 3d), and the three carrying capacities were significantly higher than those of the lower edge of the lamina (*P* < 0.05, Table 3). The maximum load of the upper and lower edges of the spinous process was significantly lower than that of each group (*P* < 0.05, Table 3), but the upper edge of the spinous process was greater than its lower edge (*P* < 0.05).

For the ultimate strain, the inferior articular process had the maximum strain (Fig. 3b), and no significant difference was found between the superior and inferior articular processes (*P* > 0.05, Table 3) but was significantly higher than the upper and lower edge of lamina (*P* < 0.05, Table 3); there was no significant difference between the superior articular process and upper edge of the lamina group (*P* > 0.05), but greater than the lower edge of the lamina (*P* < 0.05, Table 3). No significant difference was found between the upper and lower edges of the lamina (*P* > 0.05, Table 3). The spinous minimum limit strain was significantly lower than that in each group (*P* < 0.05, Table 3).

## Discussion

Each point of the rear lumbar bone structure may become an anchor point for a posterior attachment device. However, the difference in stiffness and strength of these anchor points is still poorly understood. No previous report was found regarding the mechanical properties of these anchor points measured with compression testing. In this study, we used the traditional and conventional compression platen methods [17] with a universal testing machine to perform uniaxial compression testing directly on specimens. This method is simple, and data are reliable. Our results indicated that different parts of the rear lumbar spine bone structure exhibited significant differences in mechanical properties, which could provide useful reference data regarding the best lumbar rear mooring points.

The most important information for evaluating the merits of a material is whether the material has high rigidity (stiffness) and compressive strength (strength). Our results showed that the axial pressure of the upper and inferior articular process group was 1170.79 ± 247.59 N and 1039.14 ± 419.97 N, respectively, suggesting that stress values of the articular process were significantly higher than those of the lamina and spinous processes, which strongly confirmed that the facet joints have an important bearing function on the spine mechanics.

In the lumbar motion segment, the three-joint complex consisted of the adjacent upper and lower vertebrae, front disc, and upper and lower facet joints that are located on both sides of the rear column [18]. The complex plays an important role in the spine mechanics transfer and guides the limited free movement of the lumbar spine, where the facet joints (lumbar facet joint) were second only to the front lumbar intervertebral joints for bearing stress and serving a stabilizing role. However, the facet joint determines its specific mechanical properties due to its large variation [19]. Our results theoretically and directly proved that the facet joint has an important bearing function on the mechanical properties, and this is consistent with conclusions from many previous studies [20].

In the comparative analysis of mechanical properties, we compared the upper edge of the lamina with the upper articular process and the lower edge of the lamina with the lower articular process. With a comprehensive comparison, the upper and lower articular processes had greater intensity with a strong ability to withstand compression loads, whereas the upper and lower edges of the lamina had high hardness with a strong ability to resist external deformation, which can be used as an ideal anchor point for rear lumbar attachment device.

An intriguing finding of this study is that the strength and stiffness of the spinous process, as the rearmost bone structure of lumbar column, were significantly lower than other bone parts by the comparative statistical analysis. In the design of the tension band interspinous device, Golish et al. [21] measured the carrying capacity of the L4 spinous process as 453 ± 16 N when the center line was not reduced, and after depressing, the carrying capacity decreased to 264 ± 99 N; the carrying capacity of the L5 spinous process was 517 ± 190 N. When the midline was not reduced, the carrying capacity decreased to 269 ± 184 N after depressing. When the spinous process of the lumbar spine was loaded with clamps and hooks, Shepherd et al. [22] found that the carrying capacity of L3 and L4 spinous processes was 339 N. The *N* values of the spinous process measured in our test were smaller than those in other reports, which may be caused by bone loss with time during storage because the bone strength was significantly and positively correlated to the bone mineral density. However, in our study, specimens of each group were collected from the same conditions, and the experimental results by statistical analysis indicated that the stiffness and strength of the upper and lower edges of the spinous process were significantly weaker than those of the lamina and articular processes. This strongly confirmed that the spinous process was not the best anchor point for an attachment device and that the interspinous attachment device used for posterior lumbar non-fusion could not effectively achieve spinal decompression on other rear bone parts.

## Conclusions

Compared with other bone anchor points, the upper and lower edges of the lamina have good mechanical properties, and the upper and lower articular processes have greater strength than the other parts. The mechanical properties of the spinous process were not relevant among those bone anchor points. Our data can be used for future biomechanics research on the spine and can provide a theoretical reference for improving biomechanical compatibility of the implant material.

## Abbreviations

MI-PLIF: Minimally invasive posterior lumbar interbody fusion
PLIF: Posterior lumbar interbody fusion
TLIF: Transforaminal interbody fusion
